# NFATc1 Regulation of Dexamethasone-Induced *TGFB2* Expression Is Cell Cycle Dependent in Trabecular Meshwork Cells

**DOI:** 10.3390/cells12030504

**Published:** 2023-02-03

**Authors:** Mark S. Filla, Jennifer A. Faralli, Caleigh R. Dunn, Haania Khan, Donna M. Peters

**Affiliations:** 1 Pathology & Laboratory Medicine, University of Wisconsin School of Medicine and Public Health, Madison, WI 53705, USA; 2 Ophthalmology & Visual Sciences, University of Wisconsin School of Medicine and Public Health, Madison, WI 53705, USA

**Keywords:** trabecular meshwork, TGFβ2, NFATc1, dexamethasone, cell cycle, quiescence

## Abstract

Although elevated TGFβ2 levels appear to be a causative factor in glaucoma pathogenesis, little is known about how TGFβ2 expression is regulated in the trabecular meshwork (TM). Here, we investigated if activation of the cytokine regulator NFATc1 controlled transcription of TGFβ2 in human TM cells by using dexamethasone (DEX) to induce NFATc1 activity. The study used both proliferating and cell cycle arrested quiescent cells. Cell cycle arrest was achieved by either cell–cell contact inhibition or serum starvation. β-catenin staining and p21 and Ki-67 nuclear labeling were used to verify the formation of cell–cell contacts and activity of the cell cycle. NFATc1 inhibitors cyclosporine A (CsA) or 11R-VIVIT were used to determine the role of NFATc1. mRNA levels were determined by RT-qPCR. DEX increased TGFβ2 mRNA expression by 3.5-fold in proliferating cells but not in quiescent cells or serum-starved cells, and both CsA and 11R-VIVIT inhibited this increase. In contrast, the expression of other DEX/NFATc1-induced mRNAs (myocilin and β3 integrin) occurred regardless of the proliferative state of the cells. These studies show that NAFTc1 regulates TGFβ2 transcription in TM cells and reveals a previously unknown connection between the TM cell cycle and modulation of gene expression by NFATc1 and/or DEX in TM cells.

## 1. Introduction

Glaucoma is a heterogeneous disease comprised of chronic optic neuropathies that lead to irreversible blindness due to the progressive degeneration of the optic nerve. The most common form of glaucoma in the United States is primary open-angle glaucoma (POAG). POAG is an age-related disease. During aging, there is an excessive loss of cells from the trabecular meshwork (TM) in the anterior segment of the eye. There are also structural changes to the architecture of the extracellular matrix (ECM) within the TM. Both of these changes can lead to a restriction in the drainage of aqueous humor from the anterior chamber and an increase in intraocular pressure (IOP), which can contribute to the development of POAG [1].

The cells in the TM that help to control the movement of aqueous humor through the eye consist of a mixture of smooth muscle and endothelial-like cells. Like many other cell types in adult tissues, these cells have limited replicative capacity in vivo. They are not terminally differentiated, but quiescent, since they retain the ability to re-enter the cell cycle when placed in culture [2,3].

Recent studies suggest that an elevation in the cytokine transforming growth factor beta 2 (TGFβ2) may be one causative factor for the changes observed in POAG. TGFβ2 is a pleiotropic cytokine that regulates a multitude of cellular processes, including tissue homeostasis, and has been linked to several age-related diseases [4] as well as POAG. TGFβ2 is normally found in aqueous humor at low levels where it is believed to play a role in maintaining quiescence of cells in the corneal endothelium [5,6]. Elevated TGFβ2 levels have been observed in the aqueous humor of >50% of patients with POAG [7,8,9]. Both ex vivo [10,11,12] and in vivo studies [13] have shown that elevated TGFβ2 levels lead to an increase in IOP and can cause the changes frequently observed in POAG patients. In particular, TGFβ2 causes an excessive deposition of ECM proteins in the TM including fibronectin and types I, IV and VI collagen [11]. It also increases the expression of transglutaminase, an enzyme that covalently crosslinks ECM proteins [14] that could lead to the tissue stiffening observed in glaucomatous tissues [15,16]. TGFβ2 has also been shown to trigger senescence in TM cells in culture [17], thus linking it to a possible cause for the cell loss observed in POAG. Controlling TGFβ2 expression and activity has therefore become a focal point in understanding POAG [18].

TGFβ2 expression appears to be regulated in a tissue-specific manner. For example, expression of TGFβ2 can be induced by retinoic acid in cultured keratinocytes and mouse epidermis [19]; however, during heart development, retinoic acid inhibits TGFβ2 expression [20]. Likewise, the glucocorticoid dexamethasone (DEX) inhibits TGFβ2 gene expression in chondrocytes [21], while it increases TGFβ2 protein levels in human TM cells in culture [22].

To date, however, we know very little about how TGFβ2 mRNA expression is regulated in the TM. DEX increases TGFβ2 protein levels in cultured human TM (HTM) cells without increasing mRNA levels [22]. TGFβ2 mRNA and protein levels, on the other hand, were both elevated in HTM cells overexpressing a constitutively active form of the αvβ3 integrin [23]. Since αvβ3 integrin expression and activation is increased by DEX through a calcineurin/NFATc1 pathway [24], this suggested that activation of the calcineurin/NFATc1 pathway might also be involved in regulating TGFβ2 mRNA expression.

NFATc1 belongs to a family of transcription factors that are known to play a prominent role in regulating the expression of cytokines and growth factors [25]. NFATc1 is highly phosphorylated and resides in the cytoplasm until it is dephosphorylated by the serine/threonine phosphatase calcineurin (CaN). Once dephosphorylated, NFATc1 is able to translocate into the nucleus where it partners with different transcription factors (i.e., AP1, SP1, Smad3, FOXp3 or Myc) to regulate cytokine production including expression of TGFβ [25,26]. Interestingly, activation of the CaN/NFATc1 pathway occurs when intracellular calcium levels (iCa2^+^) are elevated. Under physiological conditions, iCa2^+^ levels can be elevated by nongenomic effects elicited by glucocorticoids such as DEX [27,28], suggesting that activation of the CaN/NFATc1 pathway by DEX could regulate TGFβ2 mRNA expression in the TM.

In this study, we investigated if NFATc1 could play a role in DEX-induced TGFβ2 mRNA expression and whether that was dependent upon the proliferative state of the cell. The study used a quiescent HTM cell model that mimics in vivo conditions as well as proliferating HTM cells. Using DEX to activate NFATc1 in HTM cells, we show that activation of CaN/NFATc1 induces expression of TGFβ2 mRNA, but, surprisingly, only in proliferating cells and not in quiescent cells. This implies that TGFβ2 expression is cell cycle dependent and that the quiescent cells typically found in vivo [29,30] would not increase TGFβ2 mRNA expression in response to DEX. This is in contrast to the DEX-induced expression of other genes in HTM cells such as myocilin and the β3 integrin subunit that are cell cycle independent. This suggests that it is important to consider the proliferative state of TM cells when analyzing how signaling pathways in vitro contribute to the pathogenesis of glaucoma.

## 2. Materials and Methods

### 2.1. Cell Culture

HTM cells were established from cadaver eyes obtained from 4 donors aged 25- (N25TM-10), 27- (N27TM-2), 27- (N27TM-6) and 35- (N35TM-11) years-old in accordance with the tenets of the Declaration of Helsinki, as previously described [31,32]. The donors had no known history of ocular disease. The cells were characterized to be HTM cells based upon criteria previously described [33]. All cells were used between passages 5–8. All stocks were established and maintained in growth medium consisting of low glucose DMEM supplemented with 15% fetal bovine serum (FBS; Atlanta Biologicals, Minneapolis, MN, USA), 2% l-glutamine (Millipore-Sigma, St. Louis, MO, USA), 1% amphotericin B (Corning-Mediatech, Manassas, VA, USA), 0.05% gentamycin (Millipore-Sigma) and 1 ng/mL fibroblast growth factor 2 (FGF-2; Peprotech, Cranbury, NJ, USA). Donor information was deidentified prior to use, and use of tissue was considered exempt by the University of Wisconsin Madison Institutional Review Board.

### 2.2. Induction of TGFβ2 with Dexamethasone and Effects of NFATc1 Inhibitors

To study TGFβ2 mRNA expression in proliferating HTM cells, HTM cells were plated at a cell density of 2.3 × 10^3^ cells/cm^2^ in 60 mm dishes. The next day, cells were treated for 3 days in medium containing either 500 nM DEX or 0.1% EtOH (vehicle) in low-glucose DMEM supplemented with either 1% or 10% FBS, 2% l-glutamine, 1% amphotericin B and 0.05% gentamycin. Fresh media containing either 500 nM DEX or 0.1% EtOH was added after 48 h. After 72 h, cells were harvested for RNA analysis. In some experiments, DEX- and EtOH-treated cells were also treated at the same time with the NFATc1 inhibitors cyclosporin A (10µM; CsA, Millipore-Sigma, #C3662) or 11R-VIVIT peptide (30 µM; VIVIT, Abmole Bioscience, Houston, TX, USA, #M1261) or 0.1% vehicle (DMSO). All samples were run in triplicate and averaged.

To study TGFβ2 mRNA expression in quiescent cell cultures, fully confluent cultures of HTM cells grown in 60 mm dishes were maintained and fed daily for an additional 7 days after reaching confluency with low-glucose DMEM supplemented with 15% FBS, 2% l-glutamine, 1% amphotericin B, 0.05% gentamycin and 1 ng/mL FGF-2. After 7 days, cells were switched to media containing either 10% FBS or 1% FBS without FGF-2 and treated for 72 h with either 500 nM DEX or 0.1% EtOH. Fresh media containing either DEX or EtOH were added after 48 h. After 72 h, cells were harvested for RNA analysis. Briefly, cells were tryspinized and collected by centrifugation and then washed once with PBS. The PBS was removed, and the cell pellet was frozen at −20 °C until RNA isolation (see below). All samples were run in triplicate and averaged.

### 2.3. RNA Isolation and RT-qPCR

Total RNA was isolated using RNeasy Plus Mini Kit (Qiagen Inc., Germantown, MD, USA) according to the manufacturer’s instructions. RNA was reverse transcribed into cDNA using the high-capacity cDNA reverse transcription kit (Applied Biosystems, Waltham, MA, USA) according to the manufacturer’s instructions. RT-qPCR was performed using an Applied Biosystems QuantStudio 6 Pro Real-Time PCR system and PowerUp SYBR green master mix (Thermo Fisher Scientific, Waltham, MA, USA), as we described [23,34]. Fold changes in gene expression were determined using the ΔΔCt method. Data were normalized using either succinate dehydrogenase complex subunit A (SDHA) or hypoxanthine phosphoribosyltransferase 1 (HPRT1) as housekeeping genes. Primer-BLAST (https://www.ncbi.nlm.nih.gov/tools/primer-blast/ (accessed on 5 January 2023)) was used to design the primers (Table 1) which were made by IDT (Coralville, IA, USA).

### 2.4. Immunolabeling and Quantification of Cell Cycle Protein-Positive Cells

Cultured HTM cells were fixed with 4% p-formaldehyde in PBS for 20 min prior to permeabilizing with 0.5% TX-100 for 5 min. After blocking with 1% BSA in PBS, cells were labeled overnight at 4 °C with rabbit monoclonal antibody SP6 against Ki-67, rabbit monoclonal antibody R.229.6 against p21 or mouse monoclonal antibody 15B8 against β-catenin. Rabbit monoclonal antibody (EPR25A) and mouse monoclonal antibody GAL-13 were used as negative control antibodies. All antibodies were diluted in 1% BSA in PBS. Table 2 lists the specific primary antibodies and concentrations used. Cells were washed and then labeled with either Alexa 488-conjugated goat anti-rabbit IgG (A11034) or Alexa 546-conjugated goat anti-mouse IgG (A11030) for 45 min to detect the rabbit and mouse primary antibodies, respectively. Nuclei were labeled with Hoechst 33342 (H1399). Both secondary antibodies and Hoechst were from ThermoFisher Scientific (Waltham, MA, USA). Labeled cells were imaged using a Zeiss Imager.M2 fluorescence microscope (Carl Zeiss Microscopy, White Plains, NY, USA) together with Zen image acquisition software ver 3.079.

In order to quantify the percentage of cells positive for Ki-67 and β-catenin in proliferating HTM cells or Ki-67 and p21 in quiescent cells, 5 images at 20× magnification were randomly acquired. The number of cells positive for either β-catenin, Ki-67 or p21 was determined for each image and compared to the total number of nuclei per image.

### 2.5. Immunohistochemistry and Quantification of Cells in Tissue

Human donor eyes ages 17, 46, 46, 49 and 80 were obtained from the Lions Eye Bank of Wisconsin. The eyes were bisected, and the lens was removed. The anterior segments including the iris, ciliary body, TM/Schlemm’s canal system, cornea and sclera were fixed with 4% p-formaldehyde for 1–2 h, after which the anterior segments were cut into wedges (1–12 wedges/anterior segment) prior to paraffin embedding. None of the donors had a history of glaucoma. Five-micrometer sections were cut, mounted onto glass slides and deparaffinized in xylenes followed by rehydration in a series of 100–50% ethanol solutions. Antigen retrieval was performed at 95 °C in 10 mM EDTA with 0.1 M Tris buffer at pH 9.0 for 20 min. The sections were allowed to cool to room temperature and were blocked for 1 h with 1% BSA in PBS. Sections were then incubated with rabbit monoclonal antibody SP6 against Ki-67, rabbit monoclonal antibody R.229.6 against p21 or rabbit negative control monoclonal antibody (EPR25A) diluted in 1% BSA in PBS and incubated at 4 °C overnight (Table 2). The next day, sections were washed with PBS and then incubated with Alexa 546-conjugated goat anti-rabbit IgG (A11035; ThermoFisher Scientific). Nuclei were labeled with Hoechst 33342. Sections were washed with PBS and mounted with a glass coverslip using Shandon™ Immu-mount (ThermoFisher Scientific). Labeled cells were imaged using a Zeiss Imager.M2 fluorescence microscope together with Zen image acquisition software ver 3.079. The total number of nuclei positive for either Ki-67 or p21 was determined for each wedge and expressed as a percentage of the total nuclei per trabecular meshwork/inner wall of Schlemm’s canal.

### 2.6. Data Analysis

Data are presented as the mean ± SEM. Statistical comparisons were conducted using a one-way ANOVA and a posthoc Tukey HSD test, with a *p*-value < 0.05 being considered statistically significant. Relative quantification of RT-qPCR data were normalized to SDHA, HPRT1 or an average of both.

## 3. Results

### 3.1. Detection of Quiescent and Proliferating Cells In Vivo and In Vitro

**Figure 1:**
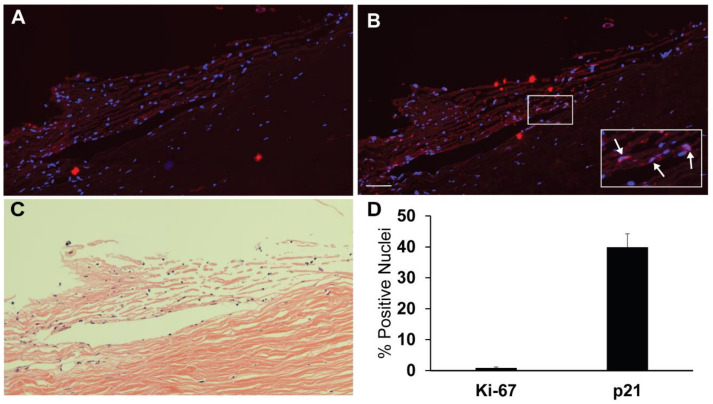
Human anterior segment labeled for proliferation marker Ki-67 and cyclin inhibitor p21. Paraffin-embedded anterior segments of normal human eyes were labeled as indicated in Methods for nuclei, Ki-67 or p21. Images shown here are representative of 5 anterior segments from different donors. (**A**) An 80-year-old female anterior segment labeled for nuclei (blue) and Ki-67 (red). (**B**) Same anterior segment labeled for nuclei (blue) and p21 (red). Enlarged inset shows nuclei positive for p21 labeling as indicated by white arrows. Scale bar = 50 um. (**C**) Hematoxylin and eosin staining of same eye as in (**A**,**B**). (**D**) Average percent of nuclei labeled with Ki-67 or p21 in the trabecular meshwork. n = 22 wedges from 5 trabecular meshworks of differently aged donors.

In order to gain a better understanding of how DEX could regulate TGFβ2 mRNA in vivo, we decided to use conditions that replicated the in vivo state of TM cells. Figure 1 shows that, as previously suggested [1], TM cells in human anterior segments are quiescent. Less than 1% of the cells showed nuclear labeling for the proliferation marker, Ki-67 [35]. In contrast, 40% of TM cells in anterior segments showed nuclear labeling for p21, a marker of cell cycle arrest and quiescence [36].

**Figure 2:**
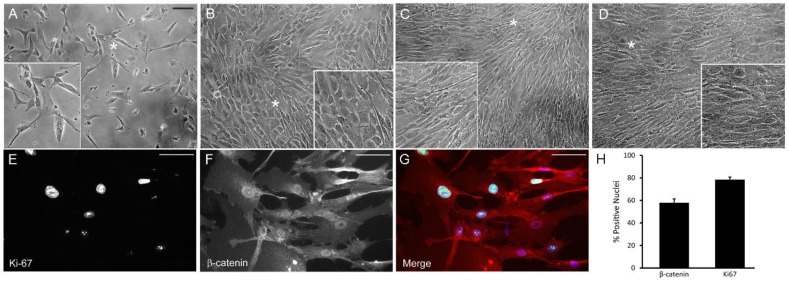
Proliferating HTM cells demonstrate nuclear labeling for Ki-67 and β-catenin. (**A**) Sparse HTM cells in culture. Bar = 100 µm. (**B**) HTM cells one day prior to reaching confluence. (**C**) HTM cells upon reaching confluence. (**D**) HTM cells 7 days post confluence. (**E**,**G**) Subconfluent HTM cells demonstrate nuclear labeling for Ki-67 (green). Bar = 50 μm. (**F**,**G**) Subconfluent HTM cells demonstrate nuclear labeling for β-catenin (red). (**H**) Quantification of the percentage of subconfluent HTM cells demonstrating nuclear labeling for Ki-67 or β-catenin. Asterisk in panels indicates area enlarged in inset.

**Figure 3:**
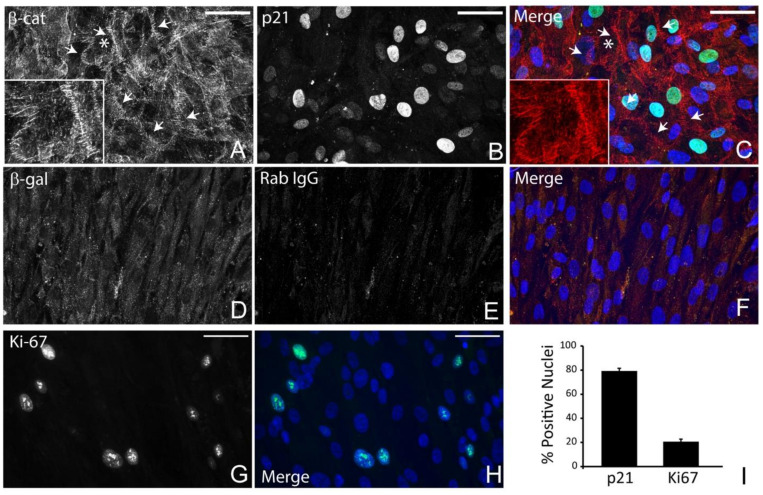
Post confluent HTM cells contain p21-positive nuclei and β-catenin-containing adherens junctions. Seven-day post confluent cultures of HTM cells labeled for β-catenin along sites of cell-cell contact (β-cat, (**A**,**C**)). Nearly 80% of the cells showed nuclear labeling for p21 (**B**,**C**,**I**), while only 20% showed nuclear labeling for Ki-67 (**G**–**I**). Nuclei are in blue (**C**,**F**,**H**). Control antibodies (**D**–**F**) are described in Methods. Bar = 50 μm. (**I**). The percentage of nuclei positive for p21 or Ki-67. Asterisk in panels (**A**, **C**) indicates area enlarged in inset.

To arrest the cell cycle and induce a quiescent state in vitro, we used cell–cell contact inhibition [37] and used cells 7 days post confluence. These post confluent cell layers have been shown to exhibit the morphological features of TM cells in vivo [3,38]. Figure 2 shows phase images of cultured HTM cells at different stages of growth. In contrast to subconfluent proliferating cells (Figure 2A) which show a more elongated appearance, HTM cells in postconfluent quiescent cultures (Figure 2D) appear densely packed and cuboidal in appearance. We analyzed subconfluent proliferating cell cultures and post confluent quiescent cells for selected cell cycle markers to see how they compared to in vivo conditions. Figure 2E–G shows nuclear labeling for Ki-67 and/or β-catenin in subconfluent proliferating cells. Like Ki-67, nuclear β-catenin localization can be used as a proliferation marker [39]. Quantification of the labeling (2H) found ~80% and ~60% of the cells demonstrated nuclear labeling for Ki-67 and β-catenin, respectively. In contrast, postconfluent quiescent cells (Figure 3) demonstrated labeling that was more like in vivo conditions. These cells did not exhibit nuclear labeling for β-catenin. Instead, they showed β-catenin labeling around the periphery of the cell (Figure 3A,C), indicating that adherens junctions, which are consistent with cells at G_0_/G_1_ in the cell cycle, had been assembled [39]. In addition, nearly 80% of the cells also showed nuclear labeling for p21 (Figure 3B,C), further indicating that the cell cycle was arrested in G_0_/G_1_ and, hence, quiescent. However, despite being cultured for 7 days post confluence, not every cell was quiescent, since the nucleus in ~20% of the cells exhibited Ki-67 labeling (Figure 3G–I).

### 3.2. DEX Induces TGFβ2 mRNA in Proliferating HTM Cells but Not Quiescent Cells

**Figure 4:**
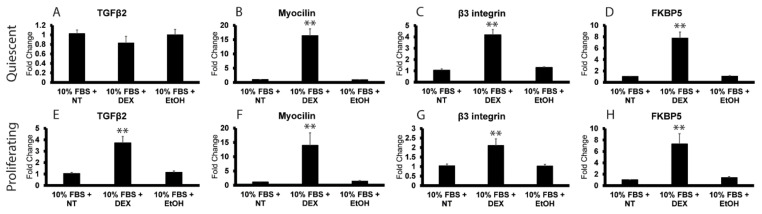
Differential DEX-induced expression of TGFβ2 mRNA in quiescent and proliferating HTM cultures. RT-qPCR analysis was used to determine mRNA levels of TGFβ2, myocilin, β3 integrin and FKBP5 in HTM cells. The names of the specific mRNAs analyzed are indicated above each panel. Panels (**A**–**D**) show 7-day post confluent cultures of HTM cells in 10% FBS treated with DEX or EtOH for an additional 72 h or left untreated (NT). Only myocilin, β3 integrin and FKBP5 increased in response to DEX. Panels (**E**–**H**) show RT-qPCR results from subconfluent cultures of the same HTM cells in 10% FBS used in panels (**A**–**D**). All genes, including TGFβ2, showed an increase in expression in response to DEX treatment. n = 5 (from 4 different cell strains). ** *p* < 0.01.

To determine the effect of DEX on TGFβ2 mRNA expression in post confluent quiescent HTM cells, the cells were treated with EtOH or DEX for 72 h. As shown in Figure 4, we did not see any change in TGFβ2 mRNA expression in DEX-treated cells in 10% FBS compared to EtOH vehicle only (Figure 4A). The cells were responsive to DEX, however, since RT-qPCR analysis showed that DEX had significantly increased expression of mRNA for the glucocorticoid response proteins myocilin and FKBP5 (Figure 4B,D) ~16- and 8-fold, respectively [24,40,41]. We also saw a 4-fold increase in β3 integrin mRNA (Figure 4C), which we previously reported was upregulated by DEX [24].

To determine if the proliferative state of the cell cycle mattered, the effect of DEX was then examined in subconfluent cultures of proliferating HTM cells that were not contact inhibited (Figure 4E–H). In contrast to what we saw in post confluent quiescent cultures, RT-qPCR analysis indicated that DEX induced TGFβ2 mRNA expression in subconfluent proliferating cells grown in 10% FBS for 72 h. As shown in Figure 4E, DEX significantly increased TGFβ2 mRNA expression by 3.5-fold (*p* < 0.01) compared to cells grown in 10% FBS in the presence of the EtOH vehicle or no treatment. RT-qPCR analysis indicated that the 72 h DEX treatment also significantly increased the expression of myocilin (Figure 4F), β3 integrin (Figure 4G) and FKBP5 (Figure 4H) mRNAs by 14-, 2- and 7-fold, respectively, compared to control groups. This suggests that the ability of DEX to induce TGFβ2 mRNA expression in HTM cells may be cell cycle dependent and that this cell cycle response is specific for TGFβ2 mRNA compared to FKBP5, myocilin and β3 integrin mRNA.

**Figure 5:**
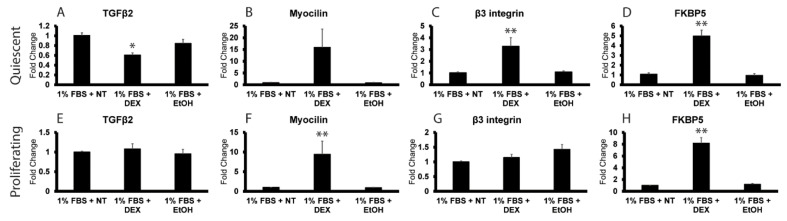
Serum starvation blocks DEX-induced expression of TGFβ2 and β3 integrin in proliferating HTM cultures. RT-qPCR analysis was used to determine mRNA levels of TGFβ2, myocilin, β3 integrin and FKBP5 in HTM cells. The names of the specific mRNAs analyzed are indicated above each panel. Panels (**A**–**D**) show 7-day post confluent cultures of HTM cells in 1% FBS that were left untreated (NT) or were treated with DEX or EtOH for 72 h. DEX increased myocilin, β3 integrin and FKPB5, but not TGFβ2 mRNA in these cells. The increase in myocilin mRNA expression, however, was not quite statistically significant compared to controls (*p* < 0.08). Panels (**E**–**H**) show that when subconfluent cultures of HTM cells in 1% FBS were left untreated (NT) or were treated with DEX or EtOH for 72 h, only mRNA levels for myocilin and FKBP5 were increased. The increases in TGFβ2 and β3 integrin mRNA levels that were observed in the presence of 10% FBS (Figure 4) were abolished in the presence of 1% FBS. n = 4 (from 2 different cell strains). * *p* < 0.05; ** *p* < 0.01.

To further establish that the expression of TGFβ2 mRNA in the presence of DEX is cell cycle dependent, the experiment was repeated in 1% FBS (low serum). Serum starvation is often used as another mechanism by which to arrest cell proliferation in vitro [42]. As shown in Figure 5, DEX failed to induce TGFβ2 mRNA expression in subconfluent proliferating HTM cells grown in 1% FBS (Figure 5E), but it did cause a 9- and 8-fold increase in myocilin (Figure 5F) and FKPB5 (Figure 5H) mRNAs, respectively. Serum starvation also prevented the DEX-induced increase in β3 integrin mRNA (Figure 5G) previously seen in subconfluent proliferating HTM cells grown in 10% serum (Figure 4).

As expected, TGFβ2 mRNA levels were not upregulated in post confluent quiescent HTM cultures grown in 1% serum; however, serum starvation had a small effect on the upregulation of β3 integrin and FKBP5 mRNAs in quiescent HTM cells. As shown in Figure 5, β3 integrin (Figure 5C) and FKBP5 (Figure 5D) mRNAs were only upregulated 3- and 5-fold, respectively, by DEX in quiescent HTM cultures in low serum compared to the 4- and 8-fold increases observed in quiescent HTM cells grown in the presence of 10% serum (Figure 4). DEX also induced expression of myocilin mRNA in quiescent cells grown in low serum, but the increase was not quite statistically significant (*p* < 0.08).

### 3.3. Reactivation of the Cell Cycle in Quiescent HTM Cells Leads to an Upregulation in TGFβ2 mRNA by DEX

**Figure 6:**
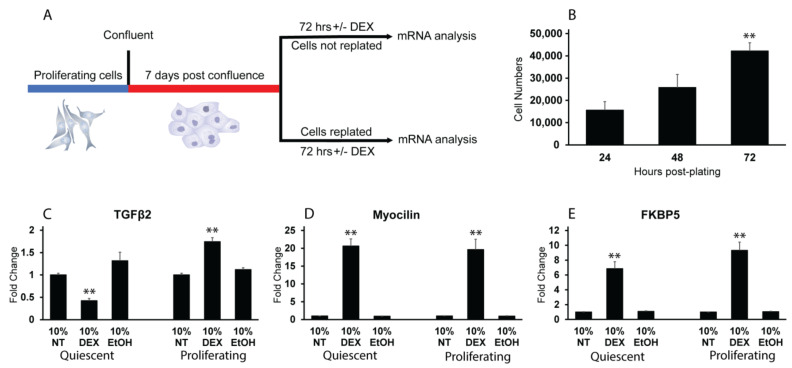
Re-entry into the cell cycle promotes DEX-induced TGFβ2 mRNA expression. (**A**). Schematic showing timeline of experiment. (**B**). Proliferative capacity of post confluent cells was verified by cell counts at 24, 48 and 72 h post replating. The 72 h time point was significantly greater than the 24 h time point, ** *p* < 0.01. (**C**–**E**). Comparison of mRNA levels for TGFβ2, myocilin and FKBP5 in 7-day post confluent cultures of quiescent HTM cells and quiescent HTM cells replated at a subconfluent density to induce re-entry into the cell cycle (proliferating). Cells were left untreated or were treated with DEX or EtOH for 72 h in the presence of 10% FBS. Quiescent cells that were forced back into the cell cycle by replating at a subconfluent density responded to DEX by increasing TGFβ2 mRNA levels in contrast to post confluent cultures that were left intact. Both groups of cells increased myocilin and FKPB5 mRNA levels in response to DEX treatment. ** *p* < 0.01, n = 2 biological replicates were conducted in triplicate.

Finally, to further demonstrate that regulation of TGFβ2 mRNA by DEX is cell cycle dependent, post confluent quiescent cells were induced to re-enter the cell cycle by dissociating cell–cell contacts. Figure 6A shows a schematic outlining the experimental protocol. Post confluent cultures were left intact or were lifted up with trypsin and replated at a subconfluent density. As shown in Figure 6B, we found that post confluent cells retained their proliferative capacity when replated. By 72 h post replating, the cells had increased their numbers by more than 4-fold over the initial plating density. We then found that when post confluent quiescent HTM cells were replated at a subconfluent density in the presence of DEX for 72 h, they showed a significant (*p* < 0.01) increase in TGFβ2 mRNA levels (Figure 6C). Additionally, they still increased both myocilin (Figure 6D) and FKBP5 (Figure 6E) mRNA levels in response to DEX under these conditions. Interestingly, DEX-treated post confluent quiescent cell cultures that remained intact and contact-inhibited showed a significant decrease in TGFβ2 mRNA (*p* < 0.01) expression instead of remaining unchanged as in earlier experiments (Figure 4). However, quiescent HTM cells still increased myocilin and FKBP5 mRNA levels in response to DEX.

### 3.4. NFATc1 Regulates Expression of TGFβ2 mRNA in Proliferating HTM Cells

**Figure 7:**
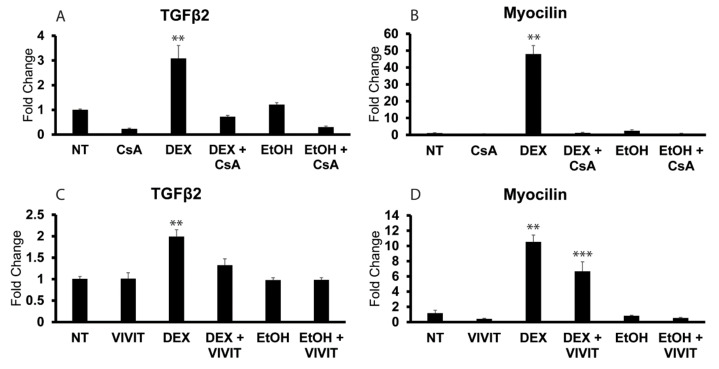
Inhibition of NFATc1 blocks DEX-induced TGFβ2 and myocilin mRNA increases in proliferating HTM cells. Subconfluent cultures of HTM cells were left untreated or were treated with DEX or EtOH for 72 h in the presence or absence of 10 µM CsA (**A**,**B**) or 30 µM 11R-VIVIT peptide (**C**,**D**). RT-qPCR analysis was used to determine mRNA levels of TGFβ2 and myocilin. Panels (**A**,**B**), TGFβ2 and myocilin mRNA levels in response to DEX only were significantly greater than NT, EtOH or DEX + CsA, ** *p* < 0.01. Panels (**C**,**D**), TGFβ2 and myocilin mRNA levels in response to DEX only were significantly greater than NT, EtOH or DEX + VIVIT, ** *p* < 0.01. DEX + VIVIT significantly > NT or EtOH, *** *p* < 0.01, n = 2 biological replicates conducted in triplicate.

Previous studies have shown that DEX-induced upregulation of myocilin and β3 integrin in quiescent HTM cells involved the CaN/NFATc1 pathway [24,41]. Since NFATc1 is known to be involved in regulating cytokine production [25], including TGFβ [26], and is also known to modulate TGFβ activity [43,44], we wanted to determine if NFATc1 activity was involved in regulating the expression of TGFβ2 mRNA in proliferating HTM cells. For this, we treated proliferating cells grown in 10% serum with either the calcineurin inhibitor cyclosporine A (CsA) or the NFATc1 inhibitor peptide 11R-VIVIT (VIVIT). As shown in Figure 7A, CsA completely blocked the DEX-induced increase in TGFβ2 mRNA levels (*p* < 0.01). It also appeared to cause a reduction in basal TGFβ2 mRNA levels seen in the no-treatment group; however, this decrease was not statistically significant. As expected, CsA completely blocked the DEX-induced expression of myocilin mRNA in proliferating HTM cells [41] (Figure 7B; *p* < 0.01). Like CsA, VIVIT also significantly reduced TGFβ2 and myocilin mRNA levels in response to DEX (Figure 7C,D; *p* < 0.01), although not to the same degree as CsA. Together, these studies suggest that the DEX-induced activation of NFATc1 activity regulates both TGFβ2 and myocilin expression in proliferating HTM cells.

## 4. Discussion

In this study, we show that the DEX-induced response in TM cells is cell cycle dependent and that actively proliferating TM cells upregulate different genes in response to DEX compared to quiescent cells. In particular, we found that only proliferating TM cells elevated TGFβ2 mRNA levels in response to DEX. This is in contrast to other DEX-responsive genes in TM cells such as myocilin and the β3 integrin subunit whose upregulation was cell cycle independent and occurred in both proliferating and quiescent cells. The regulation of TGFβ2, in part, involved the CaN/NFATc1 pathway since inhibition of CaN activity blocked its DEX-induced expression suggesting that, as was previously shown for myocilin [41], this is a secondary glucocorticoid response. In summary, this indicates that DEX has a pleiotropic effect on a number of signaling pathways in TM cells depending on the state of the cell cycle.

It should not be surprising that proliferating and quiescent TM cells respond differently to DEX. Quiescent cells exhibit a unique gene profile that is distinct from proliferating cells or senescent cells that are unable to re-enter the cell cycle [42,45]. Hence, they are programed to behave and respond to external factors differently. Furthermore, glucocorticoids have been shown to have an age-dependent effect on DNA synthesis and cell division in vivo [46], further suggesting that there would be different signaling pathways activated.

The upregulation of TGFβ2 only in proliferating TM cells may be an effort to repair and maintain quiescence in the normal adult TM in vivo [4,47]. TGFβ2 is known to play a role in arresting cell growth [48]. It is also known to suppress the immune response [4,49], thereby providing a protective function [49]. Although most of the cells in the TM in vivo are quiescent, a small percentage of TM cells exhibiting “proliferative markers” have been observed in the adult TM [1]. Why they exist is unclear. However, the cell cycle dependence of TGFβ2 expression may be a cellular strategy to not only repair the TM from age-related damages, but to return cells to a quiescent state and restore normal tissue homeostasis [50].

The cell cycle dependence of TGFβ2 mRNA expression may also be a way to prevent normal quiescent cells in the adult TM from expressing TGFβ2, since its expression can lead to senescence [4,10]. Interestingly, studies have shown that the cell cycle gene CDKN2B-AS1, an inhibitor of the cyclin-dependent kinase CDKN2B (p15^INK4b^), is associated with POAG [51], and knockdown of this gene results in an increase in TGFβ1 expression, demonstrating that control of the TGFβ pathway involves the cell cycle. Thus, there appears to be a strong link between the proliferative state of TM cells, POAG and TGFβ activity. This suggests that there is a cell cycle-dependent feedback mechanism to control TGFβ2 mRNA expression in TM cells and that a disruption in this mechanism could be responsible for POAG.

The DEX-induced upregulation of TGFβ2 and myocilin in proliferating TM cells both involved the CaN/NFATc1 pathway, suggesting that this is a common pathway involved in the DEX-induced expression of genes in the TM. It is not surprising that NFATc1 was involved in regulating TGFβ expression. Members of the NFAT family are known as important regulators of cytokine production during inflammation and have been shown to control TGFβ expression in regulatory T-cells [25]. NFATc1 has also been shown to play a role in regulating TGFβ-mediated activities [44]. How DEX activates the CaN/NFATc1 pathway requires further study.

It is unclear why the CaN/NFATc1 pathway differentially regulates TGFβ2 expression in proliferating versus quiescent cells. There could be differences in the activity of NFATc1 in proliferating and quiescent cells. For example, in some cell types, the transcriptional activity of NFATc1 is controlled by mTORC1 (mammalian target of rapamycin complex 1) [52] and/or a complex called NRON (noncoding (RNA) repressor of NFAT) [53]. Additionally, NFATc1 always partners with other transcription factors [25,26] to regulate gene expression, and it is possible that expression of one or more of these transcription factors may be influenced by the cell cycle or the presence of serum and/or DEX. This also is an area where additional studies are required.

In summary, these studies show that the upregulation of TGFβ2 mRNA by DEX in proliferating cells is a secondary glucocorticoid response that may be modulated by the CaN/NFATc1 pathway. To the best of our knowledge, this is the first time NFATc1 has been shown to regulate TGFβ2 mRNA expression outside of an immune cell. These studies also reveal a previously unknown connection between the TM cell cycle and modulation of gene expression by the CaN/NFATc1 pathway. In contrast to other DEX-responsive proteins (i.e., β3 integrin and myocilin), TGFβ2 mRNA expression was dependent on the proliferative state of the cell. This suggests that TM cells in vivo are unlikely to upregulate TGFβ2 mRNA expression during glucocorticoid-induced glaucoma unless the cells have been induced to re-enter the cell cycle. These studies show that the proliferative state of the cell cycle should be considered when studying various signaling pathways in TM cells in vitro and suggest that inhibition of specific DEX-induced responses by NFATc1 inhibitors such as CsA may be a way to alleviate side effects in response to glucocorticoid treatments.

## Figures and Tables

**Table 1:** Primers used for RT-qPCR. All sequences are given in the 5′ to 3′ direction.

Gene	Reverse Sequence	Forward Sequence
HPRT1	GGTCCTTTTCACCAGCAAGCT	TGACACTGGCAAAACAATGCA
SDHA	CACCACTGCATCAAATTCATG	TGGGAACAAGAGGGCATCTG
ITGB3	TTCTTCGAATCATCTGGCC	GTGACCTGAAGGAGAATCTGC
TGFB2	CCTCGGGCTCAGGATAGTCT	CAGCACACTCGATATGGACCA
MYOC	CTCAGCGTGAGAGGCTCTCC	GCCCATCTGGCTATCTCAGG
FKBP5	CCCTCTCCTTTCCGTTTGGTT	CTCCCTAAAATTCCCTCGAATGC

**Table 2:** Antibodies used in immunolabeling studies.

Target	Monoclonal Antibody	Host	Company	Product #	Concentration (μg/mL)
Ki-67	SP6	Rb	Abcam	ab16667	4 (cells) 5 (sections)
β-catenin	15B8	Ms	Millipore-Sigma	C7207	1
P21	R.229.6	Rb	Invitrogen	MA5-14949	1 (cells) 5 (sections)
β-galactosidase	GAL-13	Ms	Millipore-Sigma	G8021	1
KLH-conjugated small molecule (nonimmune)	[EPR25A]	Rb	Abcam	ab172730	1 (cells) 4 (cells) 5 (sections)

## Data Availability

The data presented in this study are available on request from the corresponding author.
